# Impacts of gait biomechanics of patients with thoracolumbar kyphosis secondary to Scheuermann’s disease

**DOI:** 10.3389/fbioe.2023.1192647

**Published:** 2023-05-25

**Authors:** Hao Cheng, Zi-Ang Jiang, Liang Chen, Guo-Dong Wang, Xiao-Yang Liu, Jian-Min Sun, Tsung-Yuan Tsai

**Affiliations:** ^1^ Department of Spine Surgery, Shandong Provincial Hospital, Cheeloo College of Medicine, Shandong University, Jinan, Shandong, China; ^2^ School of Biomedical Engineering and Med-X Research Institute, Shanghai Jiao Tong University, Shanghai, China; ^3^ Department of Orthopaedic Surgery, Shanghai Ninth People’s Hospital, Shanghai Jiao Tong University School of Medicine, Shanghai, China; ^4^ Department of Sports Science, Nanjing Sport Institute, Nanjing, China; ^5^ Department of Spine Surgery, Shandong Provincial Hospital Affiliated to Shandong First Medical University, Jinan, Shandong, China

**Keywords:** thoracolumbar kyphosis, gait analysis, joint angle, spatiotemporal parameters, Scheuermann’s disease

## Abstract

**Introduction:** Thoracolumbar kyphosis (TLK) is a common feature in patients with spinal deformities. However, due to limited studies, the impacts of TLK on gait have not been reported. The objective of the study was to quantify and evaluate the impacts of gait biomechanics of patients with TLK secondary to Scheuermann’s disease.

**Methods:** Twenty cases of Scheuermann’s disease patients with TLK and twenty cases of asymptomatic participants were recruited into this study. And the gait motion analysis was conducted.

**Results:** The stride length was shorter in the TLK group compared to control group (1.24 ± 0.11 m vs. 1.36 ± 0.21 m, *p* = 0.04). Compared to control group, the stride time and step time were more prolonged in the TLK group (1.18 ± 0.11s vs. 1.11 ± 0.08 s, *p* = 0.03; 0.59 ± 0.06 s vs. 0.56 ± 0.04 s, *p* = 0.04). The gait speed of the TLK group was significantly slower than it of control group (1.05 ± 0.12 m/s vs. 1.17 ± 0.14 m/s, *p* = 0.01); In the sagittal plane, the range of motion (ROM) of the hip in the TLK group was significantly smaller than that of the control group (37.71 ± 4.35° vs. 40.05 ± 3.71°, *p* = 0.00). In the transverse plane, the adduction/abduction ROMs of the knee and ankle, as well as the internal and external rotation of the knee, were smaller in TLK group than ROMs in the control group (4.66 ± 2.21° vs. 5.61 ± 1.82°, *p* = 0.00; 11.48 ± 3.97° vs. 13.16 ± 5.6°, *p* = 0.02; 9.00 ± 5.14° vs. 12.95 ± 5.78°, *p* = 0.00).

**Discussion:** The main finding of this study was that measurements of gait patterns and joint movement of the TLK group were significantly lower than those of the control group. And these impacts have the potential to exacerbate degenerative progress of joints in the lower extremities. These abnormal features of gait can also serve as a guideline for physicians to focus on TLK in these patients.

## 1 Introduction

The thoracolumbar segment is an anatomically and functionally crucial spinal segment (Bernhardt and Bridwell, 1989; Chen et al., 2001; Ikenaga et al., 2007; Buchowski et al., 2008; Enad et al., 2008). Typically, the superior endplate of T10 is parallel to the inferior endplate of L2. When the angle between the two endplates is not 0°, it is considered thoracolumbar kyphosis (TLK) (Bernhardt and Bridwell, 1989; Macagno and O’Brien, 2006; Le Huec et al., 2019; Chau et al., 2021). Many diseases can cause TLK, such as degeneration, trauma, infection, and heredity (Buchowski et al., 2008; Zhongqiang Chen, 2013; Crossfield et al., 2021). The incidence of TLK in each disease varies, for example, in Scheuermann’s disease it is less than 8% (Zhongqiang Chen, 2013), in spinal tuberculosis, it is approximately 0.5%–1.5% (Sivasamy et al., 2019), and in osteoporotic vertebral fractures, TLK appears in 8% of women over 50 years and in 27% of women over 80 years (Wang et al., 2015). TLK can also be observed in different patient populations (Chen et al., 2001; Buchowski et al., 2008; Enad et al., 2008; Zhongqiang Chen, 2013; Crossfield et al., 2021). When TLK is present, it usually results in chronic low back pain (Bernhardt and Bridwell, 1989; Macagno and O’Brien, 2006; Buchowski et al., 2008; Enad et al., 2008; Zhongqiang Chen, 2013) and sagittal spinal imbalance (Bernhardt and Bridwell, 1989; Chen et al., 2001; Macagno and O’Brien, 2006; Buchowski et al., 2008; Enad et al., 2008; Chau et al., 2021), which often results in decreased quality of life in these patients. Unfortunately, some of these cases have to undergo spinal orthopedic surgery, which brings a heavy financial and medical burden to the patient’s family (Zhongqiang Chen, 2013; Wang et al., 2015; Sivasamy et al., 2019).

Previous studies mainly focused on epidemiology, clinical treatment, and prognosis of TLK (Chen et al., 2001; Macagno and O’Brien, 2006; Ikenaga et al., 2007; Buchowski et al., 2008; Crossfield et al., 2021); however, an assessment of the TLK function is currently lacking. In addition, studies on thoracic and cervical kyphosis with gait analysis of patients’ gait parameters have indicated that stride length, stride speed, and joint angle had a strong correlation with local kyphosis (Miura et al., 2020; Asada et al., 2022; Miura et al., 2022). By analyzing the gait parameters with the same method, the effect of TLK on functional disorders can be quantified. Therefore, the basis for clinical intervention can be further provided for secondary changes in lower limb joints. Although it has been reported that patients with TLK have decreased walking ability (Miyakoshi et al., 2015; Zhang et al., 2017; Hyun et al., 2021), including walking at a slower pace, reduced walking stability, and higher chances of falling (Hirose et al., 2004; Sinaki et al., 2005; Schmid et al., 2016; Haddas et al., 2018; Miura et al., 2020), the gait pattern of Scheuermann’s disease patients with pathological TLK and the biomechanical impacts of TLK on patients’ gait remain unclear. At the same time, clinical intervention for TLK is also unknown.

The purpose of this study is to clarify the following points with gait analysis: 1) describe the gait characteristics of young patients with TLK by performing 3D gait analysis of Scheuermann’s disease patients with TLK and asymptomatic controls; 2) quantify and evaluate the secondary atypical changes in the temporal and spatial parameters of the lower extremity joints during walking in these patients by using 3D gait analysis; and 3) examine the lower extremity joint angles during the gait cycle and compare the results between the two groups, which will help researchers evaluate the difference between them and make sure whether further clinical intervention is needed.

## 2 Materials and methods

### 2.1 Selection of patients

Since TLK is relatively common in Scheuermann’s disease and the onset of TLK is rather early (Zhongqiang Chen, 2013; Hyun et al., 2021), patients in the TLK group were considered typical cases. Therefore, patients with Scheuermann’s disease combined with TLK were recruited in this study. Based on the levels of evidence from evidence-based medicine in the relevant literature, this study is a case–control study (Burns et al., 2011).

The following inclusion–exclusion criteria were used for selecting participants with TLK caused by Scheuermann’s disease:1) the patients were diagnosed with Scheuermann’s disease and complicated with TLK deformity. 2) The patient was aged between 14 to 30 years. 3) The patient’s body mass index (BMI) was less than 35. The characteristics of participating subjects are depicted in Table 1.

**TABLE 1:** Comparison of basic information (age, sex, height, weight, and BMI) of subjects between the two groups and the average Cobb angle of the TLK group.

Characteristic	Total sample (N = 40)	Group TLK	Group control	P
		(N = 20)	(N = 20)	
Age (years)	24.3 ± 3.5	24.1 ± 3.5	24.5 ± 3.5	0.719
Sex (m/f)	26/14	13/7	13/7	N/A
Height (cm)	173.9 ± 7.5	173.0 ± 6.7	174.8 ± 8.2	0.434
Weight (kg)	74.1 ± 18.1	73.7 ± 21.4	74.5 ± 14.6	0.898
BMI (kg/m^2^)	23.3 ± 5.3	22.4 ± 6.8	24.2 ± 3.2	0.290
Cobbs’ angle of kyphosis ^(o)^	N/A	15.1 ± 7.0	0	N/A

**FIGURE 1:**
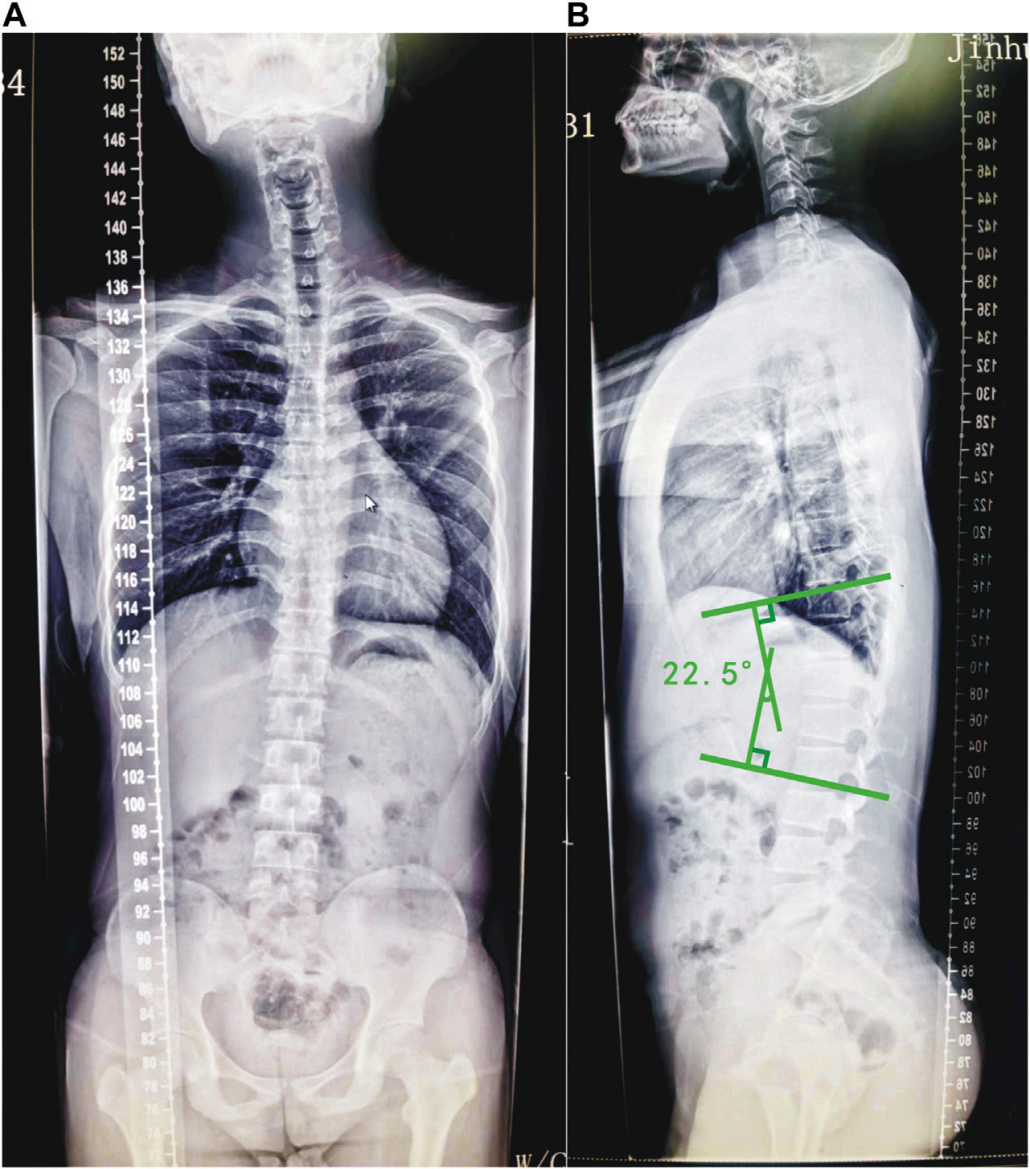
Radiograph of a typical selected example of Scheuermann’s disease patients with TLK. The anterior–posterior **(A)** view of the full spine of the patient and the lateral **(B)** view of the full spine of the patient. Draw a parallel line along the upper endplate of the T10 vertebra and a parallel line along the lower endplate of the L2 vertebra. Then, make two lines that are vertical to the aforementioned two lines. The angle between the two vertical lines is Cobbs’ angle. The Cobbs’ angle of the patient’s thoracolumbar segment is 22.5°.

A total of 20 Scheuermann’s disease patients with TLK and 20 cases of asymptomatic participants were recruited in this study. In the patient group, the mean age was 24.1 ± 3.5, whereas the mean age of the control group was 24.5 ± 3.5 (Table 1). Participants of both groups received spinal full-length anteroposterior and lateral radiographs (Philips TH-VS Dr) to determine compliance with the inclusion criteria (Macagno and O’Brien, 2006; Enad et al., 2008) prior to their participation in the study (Figure 1). The angle between the parallel line of the upper-end plate of T10 and the parallel line of the lower-end plate of L2 is Cobbs’ angle (Caesarendra et al., 2022). The Cobbs’ angle of the thoracolumbar segment on each patient’s lateral radiographs was measured using Surgimap software (Nemaris, Inc.). The mean Cobbs’ angle of the patients’ thoracolumbar segments was 15.1° ± 7.0°. The gait analysis model used Visual3D software (Visual3D, C-Motion, Inc., United States) and contained 7 bony segments, 6 joints, and 18 degrees of freedom.

### 2.2 Procedures

**FIGURE 2:**
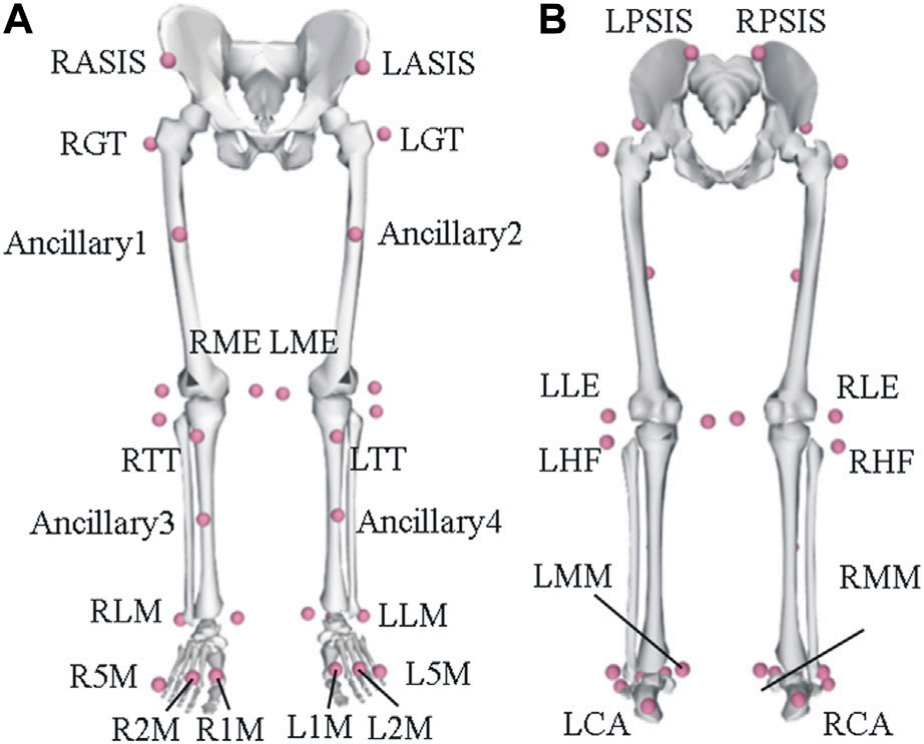
Schematic illustration of the marker set. **(A)** Anterior view of the marker placement and **(B)** the posterior view of the marker placement.

Each participant appropriately exposed the skin surface for marking the pelvis, thighs, shanks, and foot segments. A total of 32 infra-ray reflective markers were placed according to the bony landmarks as follows: bilateral anterior superior iliac spine (ASIS), posterior superior iliac spine (PSIS), great trochanter (GT), lateral epicondyle (LE), medial epicondyle (ME), the head of the fibula (HF), tibial tuberosity (TT), the lateral prominence of the malleolus (LM), the medial prominence of the malleolus (MM), the Achilles tendon insertion on the calcaneus (CA), and the dorsal margins of the first (1M), second (2M), and fifth (5M) metatarsal heads (Figure 2). Four other markers were also attached on bilateral thighs and shanks in case of the loss of markers. The principle of locating and analyzing the markers was referred to in the study by Fukuchi et al. (2018). Every marking position was manually located for accuracy on the skin by an experienced spine surgeon, and a 14-mm infrared-reflective marker was firmly attached to the location.

**FIGURE 3:**
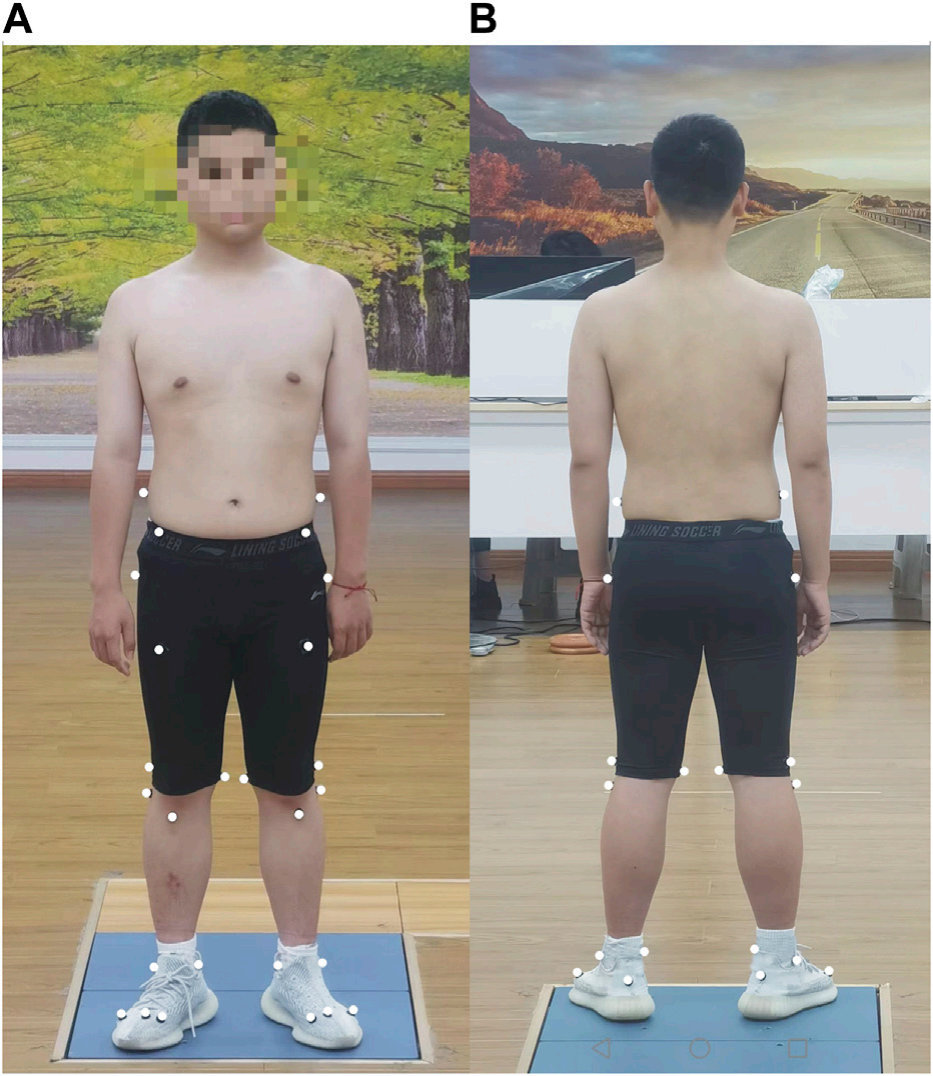
Photographs of a typical selected example of Scheuermann’s disease patients with TLK before the gait measurement begins. Anterior **(A)** view of the patient with infra-ray reflective markers on the pelvis and lower extremities. Lateral **(B)** view of the patient with infra-ray reflective markers on the pelvis and lower extremities.

All gait trials were recorded in the same gait laboratory. All participants were asked to perform walking tests at a self-selected comfortable speed (Weiner et al., 1992) in a walkway environment that was 10 m in length and 2 m in width (Figure 3). Before the experiment began, all subjects were asked to perform gait cycles several times to become used to the environment and perform natural gait states. During the experiment, each subject was asked to perform gait cycles five times, and the final result was based on the average of three valid trials. Three-dimensional (3D) kinematic data were recorded at 100 Hz using a 12-optical camera motion capture system (Vicon motion systems LTD. unit 6, Vero v2.2, Oxford, United Kingdom) in 100 Hz. In this study, all kinematic data were low-pass filtered using a fourth-ordered Butterworth filter with a cutoff frequency of 10 Hz (Leardini et al., 2007). Two AMTI force plates (BP400600, AMTI, Watertown, MA 02472, America) were also used in 1,000 Hz data-collecting frequency to record the moment of footfall and toe-off, which were used to define different gait stages.

### 2.3 Gait analysis

Before the collection of each gait data, a static collection was conducted on the subjects; they were required to choose a natural posture and stand static for at least 3 s. Static data were collected and were used to mark differences during the gait to calculate joint angles. Then, a static standing anatomical calibration trial was performed before the dynamic trial started, with the participants standing still for 1 s with their arms open on their sides. After performing the trials for dynamic gait five times, measurements were taken. All the coordination system of the lower limb segments and joints was referred to the International Society of Biomechanics (ISB) recommendation (Fukuchi et al., 2018). To compensate for anthropometric variations in height and mass (Leardini et al., 2007), all parameters were scaled and normalized in Visual3D software (Visual3D, C-Motion, Inc., United States). The average of the gait parameters of the dynamic trials was used for the subsequent analyses.

The gait variables quantified in this study were step length (m), stride length (m), stride width (m), time of step, stride, stance and swing time (s), and gait speed (m/s). The definition of the parameters is given in Table 2.

**TABLE 2:** Definitions of spatiotemporal parameters.

Parameters	Operational definition
Step length	Anterior–posterior (AP) distance from the heel of one footprint to the opposite one in heel strikes
Stride length	AP distance between heels of two consecutive footprints of the same foot comprises one stride
Step width	Lateral distance from the center of one footprint to the opposite one by two consecutive footprints
Step time	Time elapsed from initial contact of one foot to the opposite
Stride time	Time elapsed between the initial contacts of two consecutive footfalls of the same foot
Stance time	Time elapsed between the initial and the last contact of a single footfall
Swing time	Time elapsed between the last contact of the current footfall and the initial contact of the next footfall of the same foot
Gait speed	Calculated by dividing the distance walked by the ambulation time

Additionally, the measured mean kinematic data were performed as curves in the time domain over all gait cycles, including the joint angle of hips, knees, and ankles in three planes (sagittal, coronal, and transverse planes). The flexion, adduction, and internal rotation were positive, while movements in the opposite directions (extension, abduction, and external rotation) were represented by negative values. The ROM of joints in gait cycles was all normalized to 100%. The maximum range of joint angles in different directions was calculated.

### 2.4 Statistical analysis

Data were analyzed using the MATLAB (MATLAB, R2020a, MathWorks Inc., MA, United States of America) program. This study analyzed the participants’ characteristics (i.e., age, height, and weight), spatiotemporal parameters (i.e., gait velocity, stride length, and step width), and kinematic data (i.e., hip, knee, ankle joint ROMs, and angular displacements during gait) of the two groups. In addition, the biomechanical differences between the two groups were distinguished by the comparison results of spatiotemporal data and kinematic data. If the data met normal distribution assumptions and equal variances, analysis of variance was used (independent sample T-test), and a non-parametric Mann–Whitney rank test was used for non-normal distribution. Data on gait parameters and functional joint angles were expressed with a mean of 95% CI, and differences were evaluated using the unpaired *t*-test. The level of statistical significance was set at 0.05.

Data on gait parameters and functional joint angles were expressed with a mean of 95% CI, and differences were evaluated using the unpaired *t*-test. In addition, ROMs in different directions in gait cycles were also calculated, defined as the mean value of the difference between the maximum and minimum of the joint angle for each set of movements. In the processing of kinematic data, the t-test was used to calculate the difference between the joint angle values of the TLK and control groups at each time point, and the level of statistical significance was set at 0.05.

## 3 Results

Before the spatiotemporal parameters of the two groups were analyzed, the characteristics of the two groups were analyzed. The results showed no statistically significant differences in age, height, weight, or BMI between the two groups. Then, the results (mean ± SD) for the spatiotemporal gait parameters of both groups were compared (Table 3). Significant differences in the majority of spatiotemporal gait parameters were observed between the two groups. The stride length of group TLK was 91.2% of that of the control group (1.24 ± 0.11 m vs.1.36 ± 0.21m, *p* = 0.04). The strides and step time in the TLK group were 106.3% and 105.4% of those in the control group, respectively (1.18 ± 0.11s vs. 1.11 ± 0.08s, *p* = 0.03; 0.59 ± 0.06s vs. 0.56 ± 0.04s, *p* = 0.04). Moreover, the periods of single-limb stance time in the TLK group were 1.36 times than those in the control group (0.68 ± 0.08s vs. 0.5 ± 0.08 s P = 0.04). Finally, the gait speed of the control group was markedly faster than the TLK group by 11.4% (1.17 m/s vs. 1.05 m/s; *p* < 0.01). However, the parameters, including the step length, step width, and swing time, did not show significant differences between the two groups.

**TABLE 3:** Mean values, standard deviations, and the *p*-values of spatiotemporal parameter comparison between the control and the TLK groups.

	Control group	TLK group	*p*-value
Step length (m)	0.65 ± 0.05	0.62 ± 0.07	0.26
Stride length (m)	1.36 ± 0.21	1.24 ± 0.11	0.04^†^
Step width (m)	0.12 ± 0.03	0.13 ± 0.04	0.31
Stride time (s)	1.11 ± 0.08	1.18 ± 0.11	0.03^†^
Step time (s)	0.56 ± 0.04	0.59 ± 0.06	0.04^†^
Stance time (s)	0.62 ± 0.1	0.68 ± 0.08	0.04^†^
Swing time (s)	0.5 ± 0.08	0.5 ± 0.05	0.86
Speed (m/s)	1.17 ± 0.14	1.05 ± 0.12	<0.01^†^

Note: The results are expressed in the form of mean ± standard deviation; † indicates a significant difference between the two groups with a *p*-value<0.05.

**FIGURE 4:**
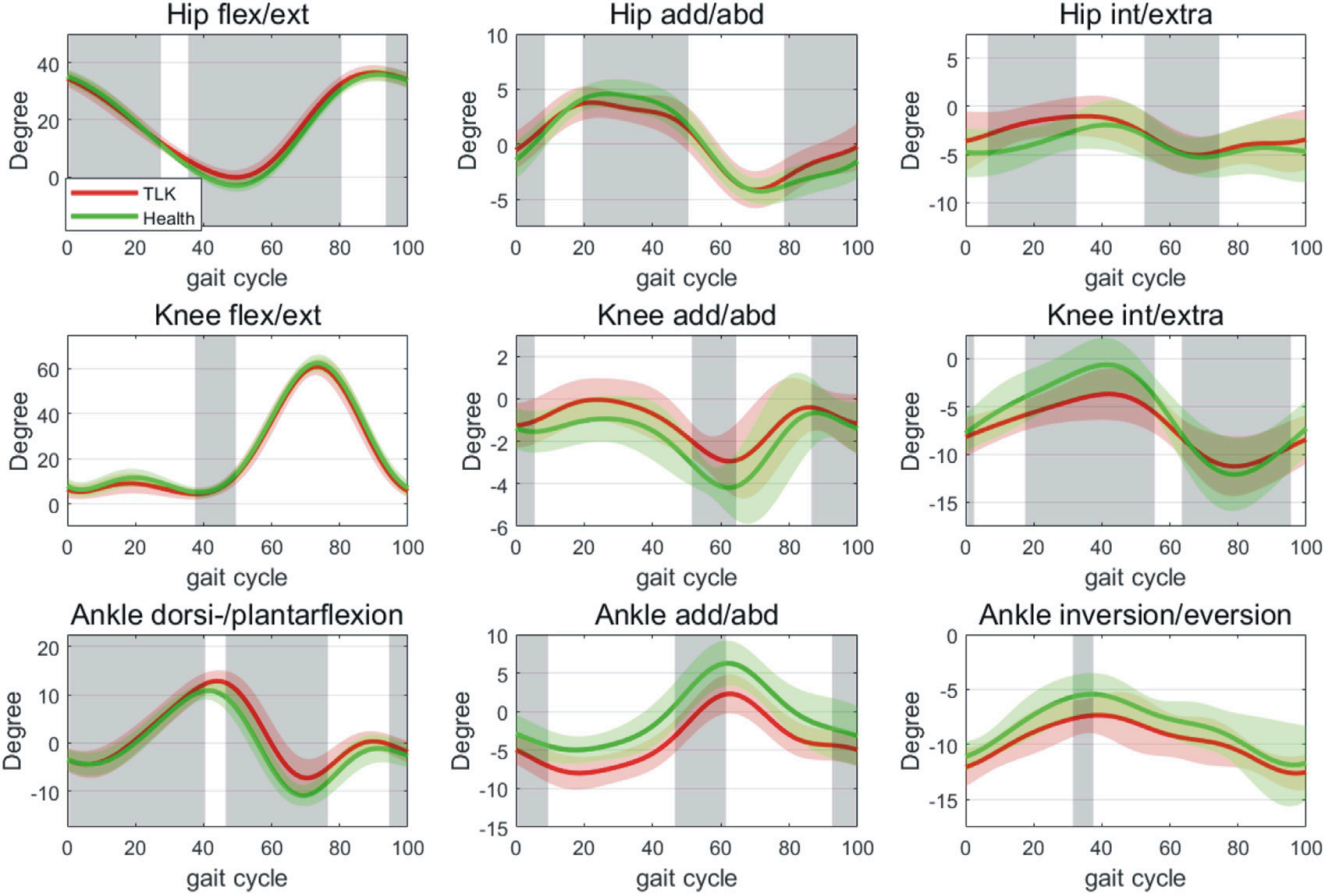
Joint-angle profiles (degrees) of hip, knee, and ankle joints across the gait cycle. The green lines and areas represent the joint angles of the control group in mean ± SD. The red lines and areas represent the joint angles of Scheuermann’s disease patients with TLK in mean ± SD. The gray areas represent the phase that showed a significant difference between the control and TLK groups in the gait cycles. As for the movement of hip, knee, and ankle joint, flexion, adduction, and internal rotation were defined as “+”, while extension, abduction, and external rotation were defined as “−”.

According to the mean curves of the joint angle progressions over gait cycles of all participants (Figure 4), significant differences in joint angles and movements were presented between the two groups. The hip joint of the control group showed a significantly higher degree of flexion in most phases (0%–26%, 93%–100%), especially in the extreme position of extension (35%–79%). In addition, the hip joint angle of the TLK group showed a significantly smaller adduction/abduction ROM around the initial contact and single support period (0%–7%, 19%–49%). In the extreme position region of the adduction, the hip joint angle of the control group was also significantly larger than that of the TLK group (78%–100%). The rotation of the hip joint in the TLK group was significantly higher in the process of rising to the first peak and the range around the trough phase (6%–31%, 52%–73%) that Scheuermann’s disease patients with TLK are more inclined to internal rotation.

The knee joint flexion/extension movement differed significantly only at the first trough position (37%–48%). In addition, the mean curve of motion showed that patients in the TLK group tended to have obvious knee adduction and exhibited significant differences around the initial contact of the start foot phase (0%–4%), along with a significantly smaller valgus peak value (51%–63%, 86%–100%). There was a substantial difference in the internal and external knee rotation, which presented a greater ROM in asymptomatic subjects at both the crest and trough of the wave (0%–1%, 17%–54%, and 63%–94%).

The ankle showed significantly greater flexion angles in the TLK group in most of the gait cycle (0%–39%, 46%–75%, and 94%–100%), especially in the second trough (46%–75%). Still, there were no significant differences in the first or second crest position. In the adduction and abduction situation, the ankle showed substantial differences around the positions of initial contact and the double support stage (0%–8%, 46%–60%), with patients of the TLK group tending to perform significant adduction of the ankle (92%–100%). However, the ankle showed no significant differences in internal/external rotation compared with the other phases except for the crest position, which showed a smaller joint rotation angle in the TLK subjects (31%–36%).

Furthermore, patients in the TLK group showed a significantly smaller hip ROM in the sagittal plane (37.71 ± 4.35 ° vs. 40.05 ± 3.71 °, *p* = 0.00); a smaller knee (4.66 ± 2.21 ° vs. 5.61 ± 1.82°, *p* = 0.00) and ankle (11.48 ± 3.97° vs. 13.16 ± 5.6 ° *p* = 0.02) ROM in the coronal plane were also performed. The knee internal and external rotation ROM of the TLK group was also smaller than that of the control group (9.00 ± 5.14° vs. 12.95 ± 5.78° *p* = 0.00) (Table 4; Figure 4). The hip ROM in the coronal and transverse planes, the knee and ankle ROM in the sagittal plane, and the ankle ROM in the transverse plane were not statistically significantly different (Table 4). In the coronal plane, the ankle showed significant differences in the positions of initial contact, double support stage, and abduction/adduction angle.

**TABLE 4:** Mean values and standard deviations of hip, knee, and ankle joints ROM in flexion/extension, adduction/abduction, and internal/external degree of freedom.

	Flexion/extension		Adduction/Abduction		Internal/external	
	Control group	TLK group	Control group	TLK group	Control group	TLK group
Hip (^o^)	40.05 ± 3.71^†^	37.71 ± 4.35^†^	9.60 ± 2.3	9.08 ± 2.61	7.02 ± 3.69	7.20 ± 3.68
Knee (^o^)	59.43 ± 5.45	59.39 ± 4.98	5.61 ± 1.82^†^	4.66 ± 2.21^†^	12.95 ± 5.78^†^	9.00 ± 5.14^†^
Ankle (^o^)	22.93 ± 3.51	22.30 ± 4.07	13.16 ± 5.6^†^	11.48 ± 3.97^†^	7.87 ± 4.77	6.83 ± 3.96

Note: The results are expressed in the form of mean ± standard deviation; † indicates a significant difference between the two groups with a *p*-value<0.05.

## 4 Discussion

This study showed that patients of the TLK group showed significant differences in gait compared to asymptomatic subjects. Due to the lack of TLK gait studies, we decided to choose the asymptomatic gait as the outcome data, which was referenced in previous studies (Chao et al., 1983; Lafond et al., 2004; Sinaki et al., 2005; Leardini et al., 2007; Hollman et al., 2011; Pataky, 2012; Miyakoshi et al., 2015; Miura et al., 2020; Kour et al., 2021).

In terms of the gait spatiotemporal parameters, patients of the TLK group showed significantly lower gait speed and longer stance time. In the sagittal plane, the ROM of the hip joint of the TLK group was significantly smaller than that of the control group, and these differences were also reflected in the joint angle diagram. As a carrier of the trunk connecting the lower limbs, the range of motion also affects the movement of the spine. Unfortunately, this study did not calculate the kinematics of the spine. However, to the best of our knowledge, this is the first study to quantify the pathological effects on the gait function in patients with TLK and we found that there may be differences in TLK gait and asymptomatic populations.

It is well-known that stride length, stride time, step time, stance time, and gait speed are all parameters related to individual ambulation (Fukuchi et al., 2018; Haddas et al., 2018). In contrast, the temporal and spatial parameters of asymptomatic subjects measured in this study showed a high degree of consistency with previous studies. In terms of step length, step width, and stride time parameters (0.65 ± 0.05 m, 0.12 ± 0.03 m, and 1.11 ± 0.08 s, respectively), they were consistent with the research results of Haddas et al. (2018) (0.60 ± 0.04 m, 0.10 ± 0.02 m, 1.14 ± 0.13 s). In addition, stride length, stance time, and swing time (1.36 ± 0.21 m, 0.62 ± 0.1 s, and 0.5 ± 0.08 s, respectively) were similar to the results measured by Konig et al. (2014) (137.64 m, 0.64 s, and 0.39 s). Moreover, the walking speed of asymptomatic subjects measured in this study (1.17 ± 0.14 m/s) was highly consistent with the results of Hollman et al. (2011) (1.12 ± 0.04 m). The comparison of the aforementioned data confirms the accuracy of the measurement methods and results in this study. As can be seen from the results (Table 3), the lower walking speed and longer stance time showed that the walking ability of patients in the TLK group was significantly decreased compared with asymptomatic participants. It is also possible that patients of the TLK group tended to take a conservative gait strategy to maintain stability and avoid falling. The trends were similar to those found in previous studies of spinal kyphotic disorders (Hass et al., 2012; Zhang et al., 2017; Miura et al., 2020). In addition, previous studies of spinal kyphotic disorders found similar findings. Moreover, it is possible that the gravity line of the trunk moves forward, which gives the trunk the tendency to move forward in the sagittal position (Fukuchi et al., 2018; Haddas et al., 2018).

In addition, it is well-known that the freedom of the hip, knee, and ankle joints in their respective motion planes is related to their own stability (Hollman et al., 2011). In a complete gait cycle, the angular kinematics results between the TLK group and the control group showed a significant difference. For example, in the sagittal plane, the hip joint showed a significantly smaller ROM during flexion (Figure 3; Table 4). This result showed similar trends and range variations along with the previous studies (Ishihara et al., 2009; Fukuchi et al., 2018; Haddas et al., 2018), which may be because the hip joint compensated to avoid falling and the excessive forward moving of the trunk gravity line caused by TLK maintained the spinal balance during gait (Le Huec et al., 2019). Another reason for the hip flexion abnormality could be chronic repetitive strain of the flexor muscle groups (psoas major and rectus femoris muscles), which connected the lumbosacral and femoral regions that kept the spinal balance in a long-term overwork situation (Xia et al., 2018; Xia et al., 2019). Consequently, this compensation may also decrease the gait speed and step length to maintain stability. In addition, ankle flexion showed a greater ROM, which may also be affected due to the compensatory decrease in hip flexion to keep the lower limbs in the relative position during gait cycles (Le Huec et al., 2019). Likewise, to maintain global balance and keep the trunk erect, patients with TLK have to increase the degree of knee flexion and ankle extension correspondingly (Matsumura et al., 2020; Chau et al., 2021; He et al., 2022). According to previous studies, long-term abnormal ROM and stress of lower limb joints, as well as a long-term strain of muscles around the joints, often lead to the emergence and aggravation of osteoarthritis (Le Huec et al., 2019). However, none of the patients in the TLK group in this study showed notable symptoms of lower limb joint osteoarthritis, which is believed to be due to the relatively young age of the patients, whose osteoarthritis was still in the early stage (Le Huec et al., 2019).

The measurement results of the lower limb joint freedom in asymptomatic participants in this study were also consistent with the previous literature reports. The results of ROM of the hip, knee, and ankle in the sagittal plane were 40.05° ± 3.71°, 59.43° ± 5.45°, and 22.93° ± 3.51°, respectively, which were almost identical to the measurements reported by Ko et al. (2011)(mean value: hip: 40.24°; knee: 55.46°; and ankle: 24.00°). Moreover, on the ROM of the coronal plane of the hip joint, the measurement of 9 .43° ± 2.55° by Haddas et al. (2018) is very similar to the measurement of 9.60° ± 2.3° in this study. However, on the coronal plane, ROMs of both the knee and ankle were lower than the measurements of Zeng et al. (2017) and Haddas et al. (2018). This may be caused by ethnic differences or different living habits.

With comparison, we observed similarities with previous studies in the variation of the joint angle graph (Ko et al., 2011; Haddas et al., 2018). In addition, in terms of the degree of freedom of internal and external rotation, the measured value of the knee joint of asymptomatic participants was 12.95° ± 5.78°, which was also relatively similar to the result reported by Zeng et al. as 11.0° ± 3.4° (2017).

Moreover, we found that the hip, knee, and ankle joints of the TLK group showed different characteristics in their respective motion planes compared with the control group. For example, patients with TLK tended to have a more limited abduction angle of the hips, especially at the crest of the first wave. The phenomenon reflected a tendency to increase the step width to keep a dynamic balance in gait performance. We know from previous studies that moderate abduction of the hip can increase its stability (Paleg et al., 2021), and the results of this study are consistent with this conclusion. Likewise, the knee and ankle movements tend to be opposite in adduction and abduction. In the freedom of adduction and abduction of knee joints, the kinematic data produced a significant difference in similar phases (Figure 3), which may allow the movement to counteract the effects from each other instead of being reflected in the spatiotemporal parameters. Meanwhile, a mutual angular adjustment among the hip, knee, and ankle joints may also exist to ensure the stability of the center of gravity in the medial–lateral direction (Le Huec et al., 2019). The mutual adjustment may further reduce the value of the angular variation difference in adduction and abduction, which leads to a step width that did not reflect the statistical significance. However, knee and ankle joint movements had significantly less freedom in the coronal plane than in asymptomatic subjects. This can be explained as the patients subconsciously becoming more careful during the gait pattern, leading to greater lower limb muscle tension because of the unconscious fear of falling (Lafond et al., 2004; Sinaki et al., 2005; Pataky, 2012; Xia et al., 2018). Ultimately, the long-term compensation of lower limbs results in the concentration of abnormal stress on the joints, which may lead to or accelerate the degeneration of joints (Matsumura et al., 2020; He et al., 2022).

In the transverse plane, the hip joint showed a tendency for more significant internal rotation (Figure 2). It showed a significant difference in the middle part of the stance phase and the early stage of the swing phase in the gait cycle, which should be the compensatory consequence of pelvic retroversion. Brunner et al. found in their research that a slight internal rotation of the hip joint can cause the pelvis to tilt forward (Brunner et al., 2008). So, to a certain extent, the phenomenon of pelvis back tilt and hip joint internal rotation is considered to be a compensatory result in response to pelvis back tilt in patients with TLK. In addition, the knee rotation of patients in the TLK group had a significantly smaller ROM than that of asymptomatic participants (Figure 3; Table 4), especially showing a limited ROM in the peak and trough of the angular kinematics data. According to Kaya Mutlu. (2018), patients with arthritis often experience joint angle restrictions. The limited ROM of the TLK group in this study may lead to abnormal stress concentrated on the medial and lateral parts of the knee joint, which, as a potential factor, may lead to the acceleration of degeneration of osteoarthritis of the knee (Herssens et al., 2018; Matsumura et al., 2020). As for the ankle joint, it shows similar motion patterns in each phase of rotation (Figure 3). Despite the pattern of knee rotation, significant differences mainly occurred in the sagittal plane of hip and ankle joints’ angular kinematic movement. We speculate that due to the appearance of TLK, the center of gravity of the body is shifted forward, followed by the retroversion of the pelvis and the corresponding compensation of the knee and ankle joints. However, the two groups were affected by differences in age, gender, Cobbs’ angle of kyphosis, and gait posture, which may lead to different gait patterns. Therefore, these factors need to be considered simultaneously in future studies.

The main finding of this study was that measurements of gait patterns and joint movement in the TLK group were significantly lower than those in the control group. For example, the walking speed of patients in the TLK group was 89.7% of that of the control group. At the same time, the ROMs of the patient’s hip, knee, and ankle joints in their respective motion plane were, respectively, 93.1%, 83.1%, and 87.2% of that of asymptomatic participants, and the knee joint in the internal and external rotation was only 69.5% of that of asymptomatic subjects. Along with the abnormal spatiotemporal parameters of patients of the TLK group during walking, the ROM and stress on patients’ lower limb joints also appear abnormal correspondingly. In the long run, osteoarthritis of lower limb joints is inevitable. However, patients of TLK did not complain of pain in lower limb joints or limited mobility. The reason may be because the patients are young and the mean degree of TLK is mild, so the abnormal stress of lower limb joints did not lead to early degeneration. However, clinicians need to pay attention to the possibility of lower limb degeneration in patients with long-term TLK.

## 5 Limitations

There are a few limitations to this study. First of all, this study only investigated the gait features in the TLK and control groups; we did not consider the potential relationship between spinal movement and gait. In addition, weakness of trunk extensors and excessive degenerative changes caused by spinal kyphosis may lead to abnormal gait characteristics (Friedrich et al., 2000; Engsberg et al., 2001; Schmid et al., 2016; Haddas et al., 2018; Miura et al., 2020). Nevertheless, the potential underlying mechanism of the relationship between the abnormality of gait posture and spatiotemporal performance has not been indicated. The correlations between kinematic and dynamic data with clinical TLK severity can be established in further intensive studies. Future studies can also investigate the outcomes of relevant treatment and rehabilitation interventions. Furthermore, we only investigated the kinematics of gait and did not deeply analyze the dynamics and muscle biomechanics of gait. Finally, the case–control study has notable limitations, which limit further exploration of the clinical conclusions.

## Data Availability

The original contributions presented in the study are included in the article/Supplementary Material; further inquiries can be directed to the corresponding authors.
